# Performance of GPT-4 for planning acupuncture treatment: comparison with human clinician performance

**DOI:** 10.3389/fmed.2025.1632303

**Published:** 2025-09-24

**Authors:** Da-Eun Yoon, Cheol-Han Kim, Yeonhee Ryu, Ye-Seul Lee, Younbyoung Chae

**Affiliations:** ^1^Department of Meridian and Acupoint, College of Korean Medicine, Kyung Hee University, Seoul, Republic of Korea; ^2^KM Fundamental Research Division, Korea Institute of Oriental Medicine, Daejeon, Republic of Korea; ^3^Jaseng Spine and Joint Research Institute, Jaseng Medical Foundation, Seoul, Republic of Korea

**Keywords:** large language model, artificial intelligence, GPT-4, medical decision-making, acupoint selection

## Abstract

**Background:**

The medical knowledge of GPT-4 has been evaluated on patient data, providing diagnostic and treatment suggestions. However, few studies have directly compared the clinical suggestions of GPT-4 with those of groups of practitioners.

**Methods:**

This study assessed the ability of GPT-4 to make medical decisions regarding acupuncture treatment by comparing its selection of acupoints with those made by human clinicians. Ten case reports published in Korean medical journals were selected and put in a standardized format. The standardized patient information was given to 80 Korean Medicine doctors and GPT-4 to diagnose and prescribe three to five acupoints per case. To evaluate the performance of GPT-4, the similarities in acupoint selection between the doctors and GPT-4 were quantified based on the percentage overlap and correlations of the selection probabilities of acupoints in each case.

**Results:**

The average percentage overlap for acupoints among cases at the 10% cutoff was 51.3%, i.e., more than half of the GPT-4 acupoint suggestions overlapped the acupoints selected by the doctors. In half of the cases, significant correlations were observed in the acupoint selection probabilities, implying that GPT-4 acupoint suggestions are similar to those of doctors.

**Conclusions:**

GPT-4 made reasonable acupoint suggestions, with notable overlap observed with the prescriptions of doctors. This shows its promise for supporting medical decisions, education, and personalized medicine for patients undergoing acupuncture treatment. Future studies and validation are necessary to ensure the reliability and efficacy of applying GPT-4 in real-world settings.

## Background

The potential of large language models (LLMs) in medicine has been demonstrated; they can contribute to medical education, research, and clinical decision-making (1). The reliability of ChatGPT, one of the first generative artificial intelligence (AI) chatbots, in medical domains has been tested since its release in 2022 by OpenAI. Its performance in medical licensing examinations in many countries and languages demonstrates its medical knowledge in both conventional and traditional East Asian medicine (TEAM) (2–5). Numerous studies have revealed its utility in medical education and as support for clinical decisions. Healthcare professionals can use ChatGPT to support clinical decisions, such as diagnosis and treatment planning, with reliable accuracy (6, 7). The benefits can be extended to education, strengthening the personalized learning experiences of students or trainees (8).

Acupuncture treatment is a clinical intervention in which the practitioner stimulates specific points on the patient's body using needles. Combinations of these points are selected according to the symptoms, diseases, and state of the patient. Selecting acupoints is a complex activity, and the selection rationale is often intrinsic; many factors affect this process, including traditional and conventional theories regarding the locations of targets as well as the experience of doctors (9, 10). With the advent of AI, data-based algorithms and models were developed to discover the rules governing acupoint selection (11, 12). Since each point has unique properties and actions, choosing the correct points is important for achieving better clinical responses (13, 14). The use of ChatGPT for suggesting acupoints has shown promise, indicating its utility in acupuncture (15).

The medical knowledge of generative AI has been evaluated using real patient cases, and it made accurate diagnoses in challenging medical cases (16). Chatbot has also suggested treatment according to patient information and provided valuable insights for tailored treatment plans (15, 17). However, few studies have directly compared the clinical suggestions of ChatGPT with those of actual practitioners. Therefore, this study assessed the practical utility of GPT-4, the latest version of ChatGPT, in making medical decisions regarding acupuncture treatment by comparing its selection of acupoints with those made by human clinicians.

## Methods

### Materials preparation

Ten cases were selected from case reports including acupuncture treatment published in Korean medical journals between 2014 and 2016. We selected ten cases to include a diverse range of diseases, covering musculoskeletal symptoms, psychiatric symptoms, and various symptoms treated in internal medicine. All patient information that could influence the diagnosis and treatment plan was extracted from each case. To standardize the information for virtual diagnosis, we organized the extracted information in the order of demographics, chief complaint, medical history, other symptoms and signs, and laboratory test results. The study was approved by the Institutional Review Board of Kyung Hee University, Seoul, Republic of Korea (KHSIRB-17-046).

### Data collection and preprocessing

**Figure 1:**
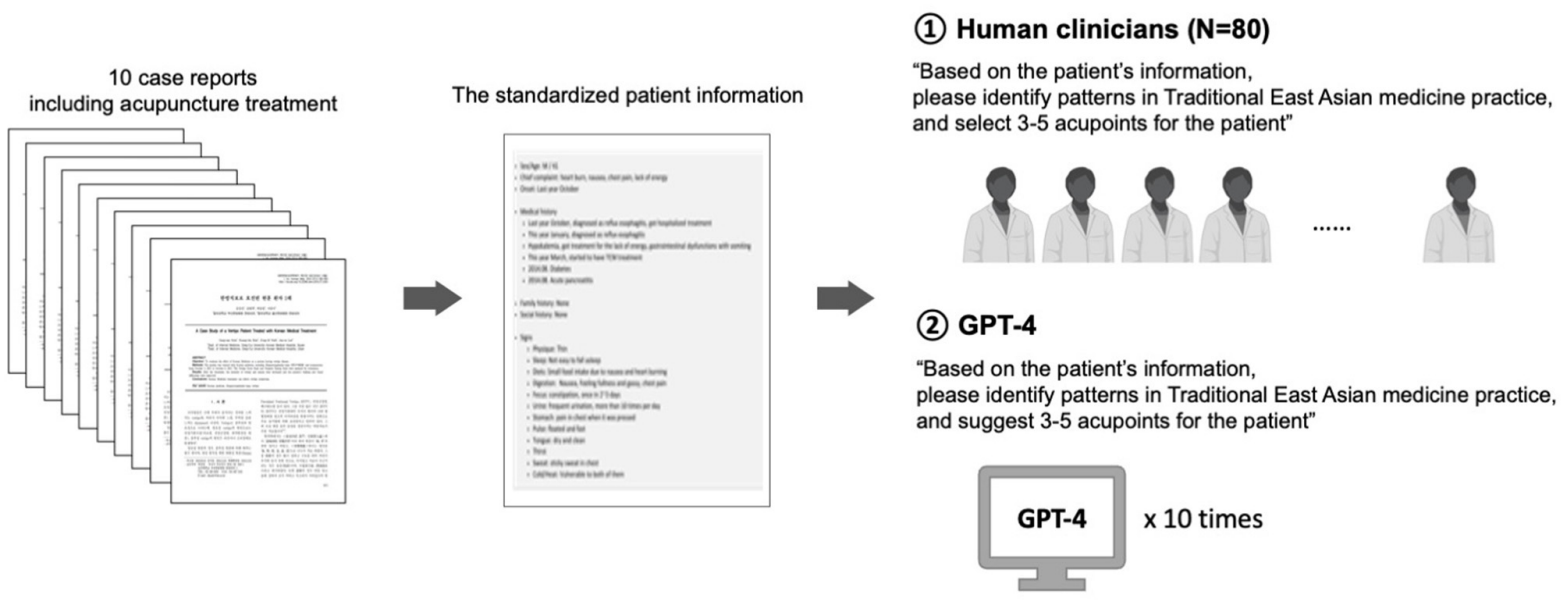
Study procedure. Ten cases were selected from case reports published in Korean medical journals. Patient information, including symptoms and signs, was standardized into a consistent format. This information was presented to 80 Korean medicine doctors, who were instructed to select three to five acupoints for each patient, and to GPT-4, which was prompted to perform the same task. KMD, average of the 80 Korean medicine doctors; GPT, average of 10 GPT-4 runs.

Through an online virtual diagnosis process, 80 Korean medicine doctors (KMDs) were provided with standardized patient information for each case. They were asked to identify TEAM patterns and select three to five acupoints based on the information (18). The patterns of acupoint selection by the doctors were previously published elsewhere (19). GPT-4 was given the same patient information, with the prompt “Based on the patient's information, please identify patterns in Traditional East Asian medicine practice, and suggest 3–5 acupoints for the patient”. The procedure was performed for all 10 cases in May 2024, and every prompt was run in independent chats to prevent any learning effects. The experimental process is described in Figure 1.

Data from 80 doctors and 10 GPT-4 runs were collected for each case. Acupoint data from doctors were initially recorded as free text, a pre-processing step was applied to correct typographical errors and standardize the data by excluding any acupoints not included in the 361 acupoints defined by World Health Organization (WHO) terminology. The GPT-4 data contained Korean, Chinese, and WHO terminology and often included minor incorrect terms. Therefore, we selectively included an acupoint only if at least two of the three correctly recorded terminologies referred to the same point. Each acupoint was converted to standard WHO terminology.

### Data analysis

The selection probability for each acupoint was calculated for both the doctors and GPT-4 by dividing the total number of answers in each case by 80 for the doctors and by 10 for GPT-4. We identified the top 30 most frequently used acupoints and visualized their selection probabilities using a heatmap. The similarity in acupoint selection between doctors and GPT-4 was evaluated using the percentage overlap of acupoints and the correlation of selection probabilities in each case. Overlapping acupoints were identified as acupoints selected at a rate above the cutoff level by both the doctors and GPT-4. The percentage overlap at the 10% cutoff was calculated as follows:


$$\begin{array}{l} \text{Percentage overlap }\left(10\%\right)=\frac{\text{KMD }\geq \;10\text{\% }\cap \text{ GPT}\text{-}4\;\geq \;10\%}{\text{KMD }\geq \;10\%}\\ \;\times 100\left(\%\right) \end{array}$$

Since the percentage overlap varies depending on the cutoff level, we calculated it at different cutoff levels ranging from 10% to 50% and then averaged the values. The resulting measure is referred to as the percentage overlap (avg.) for each case.

Spearman's correlation analysis was used to compare the selection probabilities of acupoints between doctors and GPT-4 for each case. Acupoints in each case were excluded from the correlation analyses when they were not selected by either doctors or GPT-4.

To analyze selection patterns by disease type, we categorized the ten cases into three groups: musculoskeletal symptoms (derangement of meniscus [case 4], chronic prostatitis [case 6], and intervertebral disc disorders [case 8]), psychiatric symptoms (menopausal climacteric states cases [case 3], panic disorder [case 7], and fibromyalgia [case 9]), and various symptoms in internal medicine (benign paroxysmal positional vertigo [case 1], gastroesophageal reflux disease [case 2], diabetic neuropathy [case 5], and puerperal disorder [case 10]). This categorization aligns with the clusters identified in our previous study using a hierarchical approach based on selection probabilities (19). Percentage overlap was averaged for each group.

The data analyses were performed using R software (version 4.0.2, https://cran.r-project.org), and the heatmap was generated with Orange software (version 3.36.1, https://orangedatamining.com).

### Analysis of response variability

Given the substantial sample-size imbalance (10 trials of GPT-4 vs. 80 doctors responses), we compared response variability. To quantify the response variability of GPT-4 and doctors, we represented each response as a set of selected acupoints (binary, multi-label) and computed the mean of all pairwise Jaccard dissimilarities, using the following equation:


$$\begin{array}{l} d_{J}=1-\;\frac{{|\;S}_{i}\;{\cap \;S}_{k}\;|}{{|\;S}_{i}\;{\cup \;S}_{k}\;|} \end{array}$$

This approach directly measures pattern heterogeneity between two datasets with identical item availability. To characterize uncertainty under unequal sample sizes, we reported 95% bootstrap percentile confidence intervals (2,000 resamples). To assess the dispersion of heterogeneity, we applied PERMDISP permutation test, which embeds the Jaccard distance matrix via principal coordinates analysis and compared mean distances to the group centroid.

## Results

### Percentage overlap of selected acupoints between doctors and GPT-4

Table 1 shows the percentage overlap of acupoints chosen by doctors and GPT-4. The average percentage overlap between doctors and GPT-4 at the 10% cutoff among cases was 51.3%, indicating that more than half of the acupoints suggested by GPT-4 matched those selected by the doctors. The average percentage overlap across different cutoff levels was 48.9%, and the results for each cutoff level are presented in Supplementary material 1.

**Table 1:** Summary of acupoint selection similarity between human clinicians and GPT-4.

	**Case1**	**Case2**	**Case3**	**Case4**	**Case5**	**Case6**	**Case7**	**Case8**	**Case9**	**Case10**	**Average**
Disease	Benign paroxysmal positional vertigo	Gastro-esophageal reflux disease	Menopausal climacteric states	Derangement of meniscus	Diabetic neuropathy	Chronic prostatitis	Panic disorder	Intervertebral disc disorders	Fibromyalgia	Puerperal disorder	
Group	3	3	2	1	3	1	2	1	2	3	
Overlapping ratio (10%)	42	56	70	71	67	29	73	27	47	31	51.3
Overlapping ratio (ave)	32	76	56	53	79	18	39	37	58	37	48.9
KMD Top 3 Acupoints	ST36	ST36	LI4	ST35	LI4	KI3	CV17	GV3	LI4	ST36	
	CV12	CV12	LR3	SP10	ST36	CV4	PC6	BL23	LR3	LI4	
	LI4	LI4	CV17	ST36	LR3	SP6	LR3	BL25/GB30	SP6	SP6	
GPT Top 3 Acupoints	GV20	CV12	SP6	GB34	SP6	SP6	LR3	GB30	SP6	SP6	
	GB20	LR3	GV20	ST36	LR3	GB30	HT7	GB34	BL23	CV6	
	BL18	PC6	LR3/HT7	SP10	ST36	CV3	CV12	BL23	LR3	LR3	
Spearman'sCorrelation	*ρ =* 0.086	*ρ =* 0.102	*ρ =* 0.355	*ρ =* 0.159	*ρ =* 0.532	*ρ =* 0.263	*ρ =* 0.408	*ρ =* 0.107	*ρ =* 0.262	*ρ =* 0.216	
	*p =* 0.490	*p =* 0.413	*p =* 0.003	*p =* 0.213	*p <* 0.001	*p =* 0.017	*p <* 0.001	*p =* 0.415	*p =* 0.019	*p =* 0.087	

Group 1 = diseases with musculoskeletal symptom, Group 2 = diseases with psychiatric symptom, Group 3 = diseases with various symptoms in internal medicine. Overlapping ratio (10%) = overlapping ratio between Korean Medicine doctors and GPT-4 at the 10% of cutoff, Overlapping ratio (ave) = average of the overlapping ratio between Korean Medicine doctors and GPT-4 in different cutoff levels, from 10% to 50%, KMD Top 3 acupoints = top three most frequently selected acupoints in Korean Medicine doctors, GPT Top 3 acupoints = top three most frequently suggested acupoints in GPT-4.

The percentage overlap differed by the type of disease. Generally, acupoint suggestions for musculoskeletal symptoms showed the lowest overlap, with an average of 42.3% at the 10% cutoff and 36% across various cutoff levels. In comparison, psychiatric symptoms had highest overlap, averaging 63.3% at the 10% cutoff and 51% across various cutoffs, while internal medicine symptoms showed 49% and 56%, respectively.

### Selection patterns of acupoints of doctors and GPT-4

**Figure 2:**
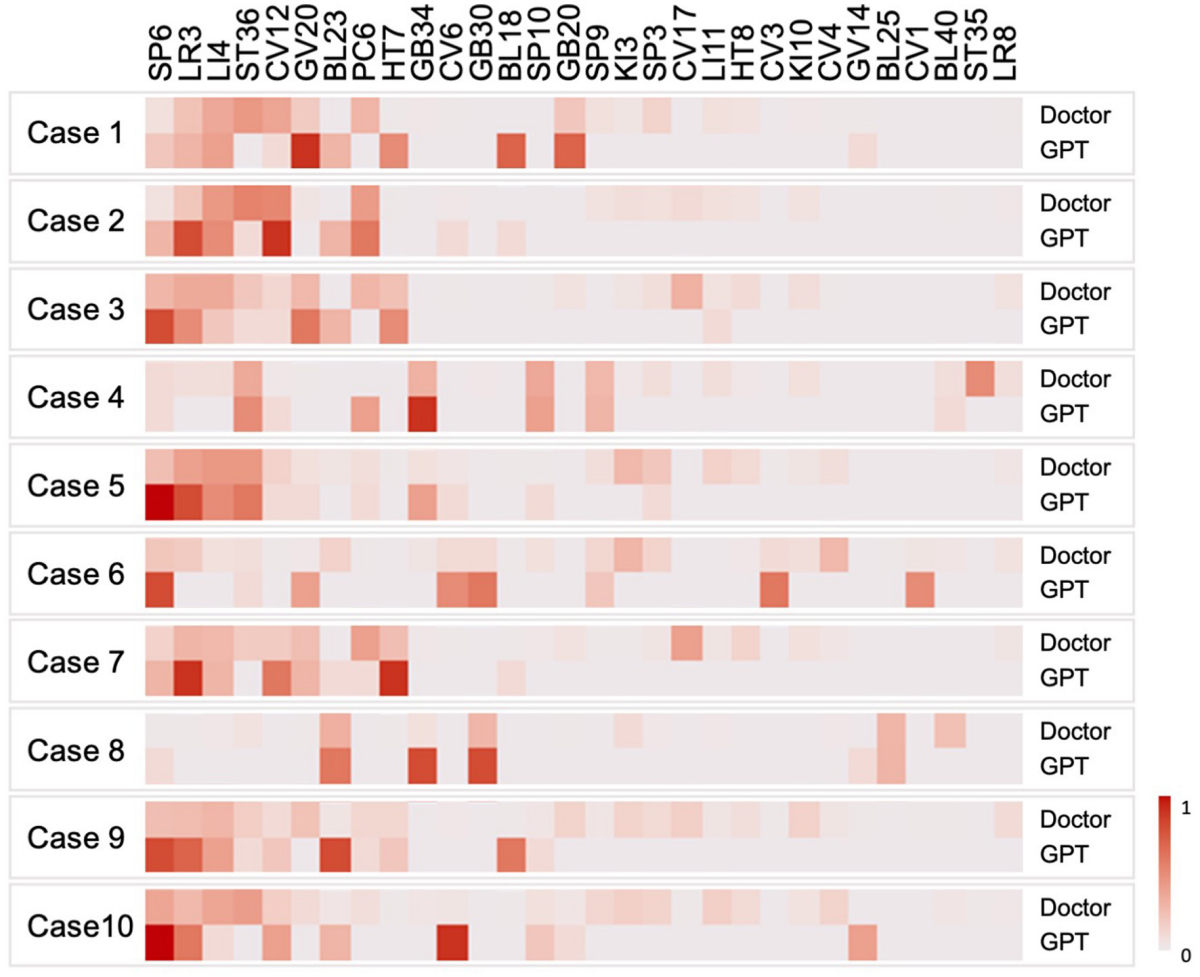
Selection probabilities of the top 30 acupoints. The selection probabilities of the top 30 most frequently used acupoints are illustrated in each of the 10 cases using a heatmap. The top row indicates the selection probabilities of the 80 doctors and the bottom row the selection probabilities of GPT-4. The color intensity in the heatmap reflects the selection probability, with darker shades indicating a higher probability of selection for a given acupoint.

The selection probabilities of the top 30 acupoints were visualized using a heatmap (Figure 2). Acupoint selection patterns were similar between doctors and GPT-4. Major acupoints such as SP6, LR3, LI4, and ST36 were consistently recommended by both doctors and GPT-4 across the 10 different diseases. Furthermore, similar acupoint selection patterns were observed beyond these major points. For example, GB20 was particularly recommended in case 1, SP10 in case 4, and BL23 in case 8.

Significant correlations were observed for 5 of the 10 cases, including case 3 (ρ = 0.355, *p* = 0.003), case 5 (ρ = 0.532, *p* < 0.001), case 6 (ρ = 0.263, *p* = 0.017), case 7 (ρ = 0.408, *p* < 0.001), and case 9 (ρ = 0.262, *p* = 0.019). These results suggest that GPT-4's acupoint selection is similar to that of doctors, as acupoints frequently chosen by doctors were also frequently selected by GPT-4, while less commonly chosen acupoints were similarly selected less often. Table 1 summarizes the acupoint selection similarities between doctors and GPT-4 for the 10 different diseases.

### Comparison of response variability between doctors and GPT-4

Across all 10 cases, doctors exhibited higher response variability than GPT-4. Mean pairwise Jaccard dissimilarity was 0.570–0.711 for GPT-4 vs 0.840–0.935 for doctors (mean difference = 0.264). Size-matched bootstrap CIs for doctors –GPT-4 excluded zero in every case, and PERMDISP likewise showed greater dispersion for doctors (all *p* < 0.05; 0.0001–0.0029). Taken together, both metrices indicate that doctors' responses are consistently more heterogeneous than GPT-4's, independent of sample-size imbalance.

## Discussion

In this study, GPT-4 showed its potential for assisting in clinical decision-making by helping to select appropriate acupoints. More than half of the acupoints suggested by GPT-4 overlapped those selected by the 80 doctors across 10 cases involving different symptoms. Moreover, significant correlations were observed in the acupoint selection patterns between doctors and GPT-4 in half of the cases. Our findings suggest that ChatGPT has the ability to suggest appropriate acupoints based on patient information and might be useful for supporting medical decisions and education regarding acupuncture practice.

We compared the acupoints suggested by dozens of doctors, and the results demonstrate how well GPT-4 can reflect real-world clinical decisions. Unlike most medical diagnoses, the selection of appropriate acupuncture points does not involve clear-cut “true” or “false” answers in most clinical settings. Therefore, we identified acupoints that were frequently suggested (those with a frequency exceeding 10%, by both GPT-4 and the doctors) and found significant overlap in these frequently suggested acupoints. Over half of the acupoints suggested by GPT-4 matched those of the doctors, with 51.3% overlap across 10 different cases. The average overlap was also similar (48.9%) when we applied different cutoff levels, indicating that GPT-4 can choose appropriate acupoints based on the clinical information of diverse patients similar to Korean Medicine doctors. Furthermore, the significant correlation of selection probabilities reveals the similarity of acupoint prescription between human doctors and GPT-4. These findings add to the growing evidence of the clinical decision-making ability of ChatGPT using patient information, such as for diagnostic reasoning and treatment planning (15, 16).

Both doctors and GPT-4 suggested major acupoints, such as SP6, LR3, LI4, and ST36, regardless of the disease. These acupoints are widely used in diverse conditions and have general neurological effects (20). One might have concerns about GPT-4 repeatedly suggesting major acupoints, as this could lead to overestimation of its performance. However, similar acupoint selection patterns were observed beyond these major points, such as GB20, SP10, and BL23, each with specific implications. For example, in the first case, GB20 was chosen to target specific symptoms and disease sites. The combination of major points for general effects and non-major points for targeted effects is commonly observed in acupuncture treatment (19). Moreover, the significant correlations in acupoint selection between doctors and GPT-4 in half of the cases further support the similarity in acupoint selection patterns exhibited by GPT-4.

The similarity of acupoint selection patterns varied by disease type. Overlap was higher, and significant correlations were more frequently observed in psychiatric disorders compared to musculoskeletal disorders. These differences may be attributed to the differing rationales behind acupoint selection depending on disease type. In musculoskeletal diseases, doctors more frequently selected local acupoints near the symptom site. While GPT-4 relies on theoretical and literature-based knowledge, doctors make decisions based on both experience and theoretical-based knowledge. Doctors might place greater reliance on their clinical experience when selecting acupoints for musculoskeletal diseases. Differences were also evident at the case level. For example, in case 1, a high percentage of doctors selected ST36 and PC6, but these were never chosen by GPT-4. Conversely, GPT-4 frequently suggested points such as BL18 and HT7, which were rarely or never recommended by doctors. Such discrepancies suggest that higher acupoint overlap does not necessarily guarantee alignment with the underlying clinical rationale of real-world clinical decisions. Caution is therefore warranted when applying language models in clinical settings.

Our study reflects the reasoning process in acupuncture treatment—where doctors integrate signs and consider multiple symptoms when selecting appropriate acupoints (9), rather than relying solely on “extrinsic” diagnoses—leads to identifiable patterns in acupoint selection. Notably, similar patterns were observed in GPT-4, suggesting that it may reflect aspects of this clinical reasoning process. Incorporating information relating to various aspects, such as subjective symptoms and individual lifestyles, could enhance personalized medicine (21). In future studies, this capability can be further leveraged by applying advanced technologies. A modified language processing model capable of handling specialized knowledge and terminology in the field of acupuncture can be utilized (22). Additionally, supervised fine-tuning can be applied to improve reliability and performance by training the model on high-quality datasets. Significant effectiveness of refined generative LLMs has been shown in the field of Traditional Chinese Medicine (23).

An enhanced version of GPT-4 could be applied to acupoint recommendation in various scenarios. A conversational AI system could be developed to allow users to input symptoms and either receive appropriate acupoint recommendations or be prompted with further diagnostic questions. However, it is important to note that, as an LLM, it cannot capture information obtained through direct in-person interaction. The process of translating such observations into text remains a task that only trained doctors can perform. Furthermore, the knowledge that LLMs learn from training data does not inherently include the experiential insights of doctors. Therefore, when applying GPT-4 to decision-making in clinical settings, careful consideration is needed to ensure that medical professionals appropriately integrate their clinical expertise. Additionally, such system could be beneficial in medical education by allowing students to input symptoms, engage in interactive discussions, and explore various approaches to acupoint selection. This could help students develop a deeper understanding of acupuncture treatment strategies while fostering a more dynamic learning experience.

Despite our findings, several limitations and challenges should be addressed regarding the application of GPT-4 in acupuncture treatment. First, this study did not apply advanced techniques for better performance, such as fine-tuning methods or prompt engineering. Although our study showed that GPT-4 performed remarkably, substantial evidence suggest that these techniques can further improve its performance in medical domain. In particular, supervised fine-tuning trains the model on selectively curated datasets, it can help address a key limitation—GPT-4 might have learned detailed clinical interventions and case-specific information from the published case reports used in this study. If GPT-4 has learned from these case reports, its responses may be biased toward the reported treatment interventions, potentially limiting its generalizability in clinical applications and hindering its broader applicability in clinical decision-making. Supervised fine-tuning methods, together with prompt engineering could help address these concerns and lead to more accurate responses in future studies. Second, the lack of representativeness and generalizability should be noted. The selected 10 cases and 80 doctors from Korea do not fully reflect acupuncture treatment practices across different cultural and educational backgrounds. Recent findings have shown that China-based models performed better on Traditional East Asian Medicine tasks (24). Moreover, relying solely on Korean clinicians and case reports may introduce inherent cultural biases, which could limit the representativeness of our findings. These factors highlight the need for future studies to expand the range of sources, clinicians, and models to improve representativeness and mitigate cultural bias. Third, GPT-4's knowledge is limited by a temporal cutoff and may not incorporate the most recent evidence or clinical guidelines. In addition, this study could not include physical examination findings from direct observation, which are critical in real-world clinical decision-making. Nevertheless, our study design ensured that both GPT-4 and doctors were provided with the same patient information and the same task request for acupoint suggestion, allowing comparison under equivalent conditions. Fourth, there was a notable sample-size imbalance (10 GPT-4 runs vs. 80 doctors' responses). Nonetheless, variability analyses showed that doctors' responses were consistently more heterogeneous than GPT-4, suggesting that 10 GPT-4 runs, while still a limitation, can reasonably represent GPT-4's outputs under fixed prompt conditions. Finally, ethical issues, the generation of inaccurate content, and issues of reproducibility and uncertainty have been raised consistently regarding the use of chatbots in healthcare (1). These risks should be considered carefully when applying and interpreting the output of chatbots in clinical practice.

## Conclusions

In conclusion, our findings provide preliminary evidence that GPT-4 may support acupoint selection, in some cases making decisions similar to those of human clinicians. This suggests that generative AI could potentially be applied to support medical decision-making as well as acupuncture education and personalized medicine. By offering recommendations comparable to those made by experienced practitioners, such models may serve as a valuable tool for enhancing clinical practice, education, and patient care in acupuncture medicine. However, larger validation studies are needed to develop a more reliable and effective tool for real-world use.

## Data Availability

The original contributions presented in the study are included in the article/Supplementary material, further inquiries can be directed to the corresponding author.
