# Supplementary material for: Performance of GPT-4 for planning acupuncture treatment: comparison with human clinician performance
Source: Front Med (Lausanne). 2025 Sep 24;12:1632303. doi: 10.3389/fmed.2025.1632303 (PMC12504477; doi:10.3389/fmed.2025.1632303)

Supplementary1. Overlapping ratios and acupoints for various cutoff levels in ten cases

1. Case 01 (Benign paroxysmal positional vertigo)

| KMD  GPT | 0.10 | 0.20 | 0.30 | 0.40 | 0.50 |
| --- | --- | --- | --- | --- | --- |
| 0.10 | 0.42 | 0.33 | 0.33 | 0.25 | 0.17 |
| 0.15 | 0.56 | 0.44 | 0.44 | 0.33 | 0.22 |
| 0.20 | 0.50 | 0.38 | 0.38 | 0.25 | 0.13 |
| 0.25 | 0.50 | 0.25 | 0.25 | 0.25 | 0.00 |
| 0.30 | 0.67 | 0.33 | 0.33 | 0.33 | 0.00 |
| 0.35 | 0.67 | 0.33 | 0.33 | 0.33 | 0.00 |
| 0.40 | - | - | - | - | - |


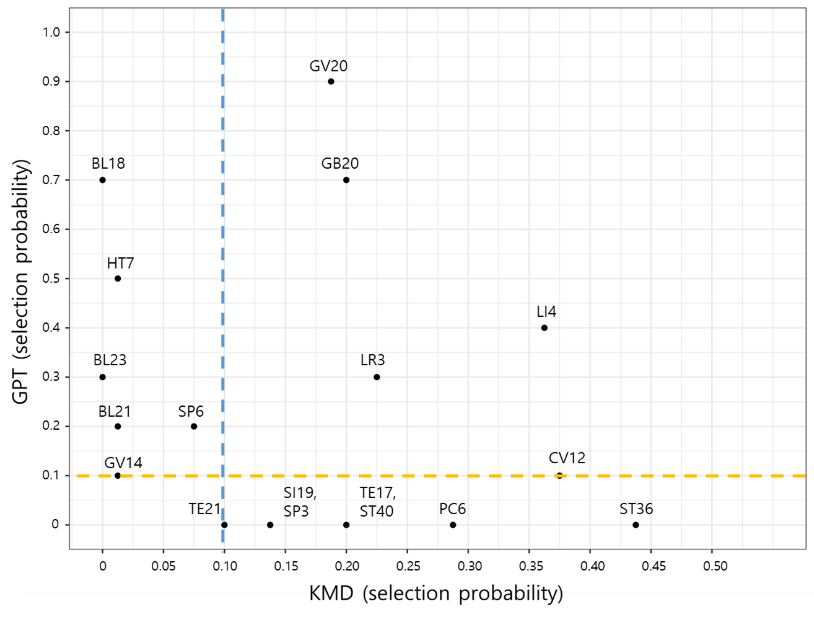


2. Case 02 (Gastroesophageal Reflux)

| KMD  GPT | 0.10 | 0.20 | 0.30 | 0.40 | 0.50 |
| --- | --- | --- | --- | --- | --- |
| 0.10 | 0.56 | 0.44 | 0.44 | 0.44 | 0.44 |
| 0.15 | 1.00 | 0.80 | 0.80 | 0.80 | 0.80 |
| 0.20 | 1.00 | 0.80 | 0.80 | 0.80 | 0.80 |
| 0.25 | 1.00 | 0.75 | 0.75 | 0.75 | 0.75 |
| 0.30 | 1.00 | 0.75 | 0.75 | 0.75 | 0.75 |
| 0.35 | 1.00 | 0.75 | 0.75 | 0.75 | 0.75 |
| 0.40 | 1.00 | 0.75 | 0.75 | 0.75 | 0.75 |


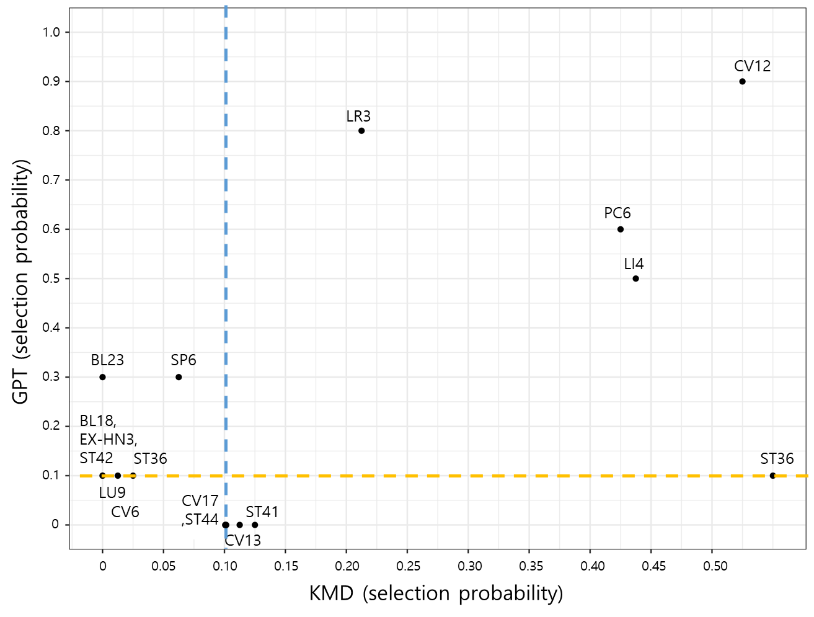


3. Case 03 (Menopausal Climacteric states)

| KMD  GPT | 0.10 | 0.20 | 0.30 | 0.40 | 0.50 |
| --- | --- | --- | --- | --- | --- |
| 0.10 | 0.70 | 0.50 | 0.40 | 0.40 | 0.40 |
| 0.15 | 0.75 | 0.63 | 0.50 | 0.50 | 0.50 |
| 0.20 | 0.75 | 0.63 | 0.50 | 0.50 | 0.50 |
| 0.25 | 0.67 | 0.67 | 0.50 | 0.50 | 0.50 |
| 0.30 | 0.67 | 0.67 | 0.33 | 0.33 | 0.33 |
| 0.35 | 1.00 | 1.00 | 0.50 | 0.50 | 0.50 |
| 0.40 | - | - | - | - | - |


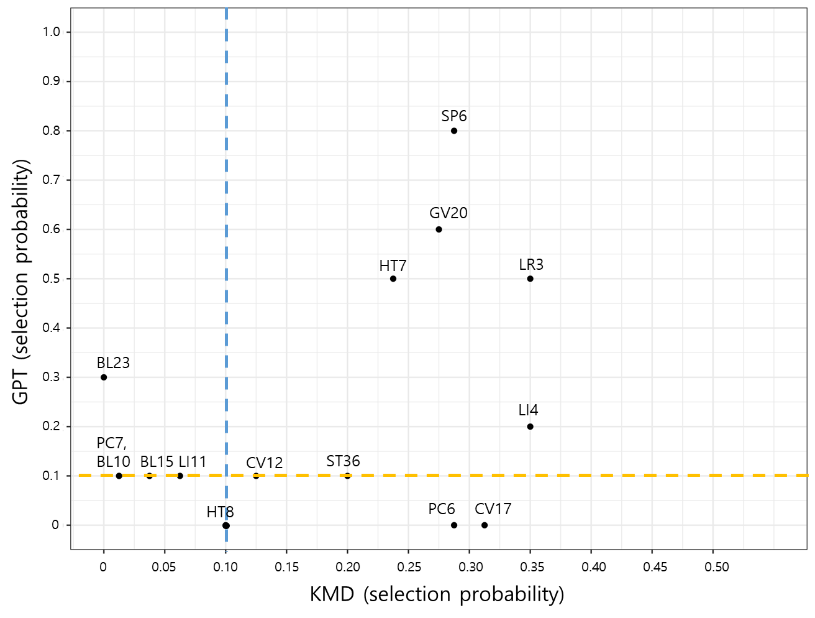


4. Case 04 (Derangement of meniscus)

| KMD  GPT | 0.10 | 0.20 | 0.30 | 0.40 | 0.50 |
| --- | --- | --- | --- | --- | --- |
| 0.10 | 0.71 | 0.57 | 0.57 | 0.43 | 0.29 |
| 0.15 | 0.67 | 0.67 | 0.67 | 0.50 | 0.33 |
| 0.20 | 0.80 | 0.80 | 0.80 | 0.60 | 0.40 |
| 0.25 | 0.80 | 0.80 | 0.80 | 0.60 | 0.40 |
| 0.30 | 0.75 | 0.75 | 0.75 | 0.75 | 0.50 |
| 0.35 | 0.67 | 0.67 | 0.67 | 0.67 | 0.33 |
| 0.40 | 0.00 | 0.00 | 0.00 | 0.00 | 0.00 |


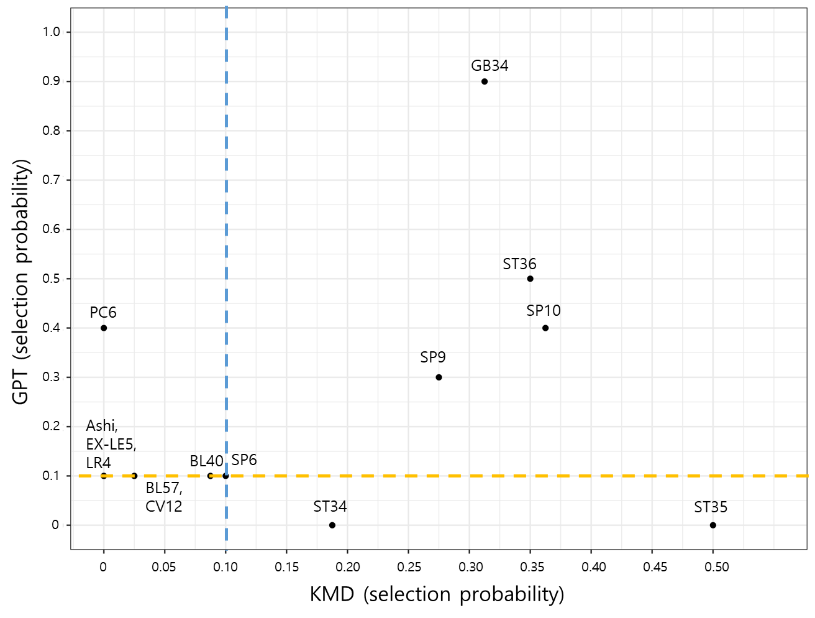


5. Case 05 (Diabetic neuropathy)

| KMD  GPT | 0.10 | 0.20 | 0.30 | 0.40 | 0.50 |
| --- | --- | --- | --- | --- | --- |
| 0.10 | 0.67 | 0.44 | 0.44 | 0.44 | 0.44 |
| 0.15 | 0.75 | 0.50 | 0.50 | 0.50 | 0.50 |
| 0.20 | 0.83 | 0.67 | 0.67 | 0.67 | 0.67 |
| 0.25 | 0.80 | 0.80 | 0.80 | 0.80 | 0.80 |
| 0.30 | 1.00 | 1.00 | 1.00 | 1.00 | 1.00 |
| 0.35 | 1.00 | 1.00 | 1.00 | 1.00 | 1.00 |
| 0.40 | 1.00 | 1.00 | 1.00 | 1.00 | 1.00 |


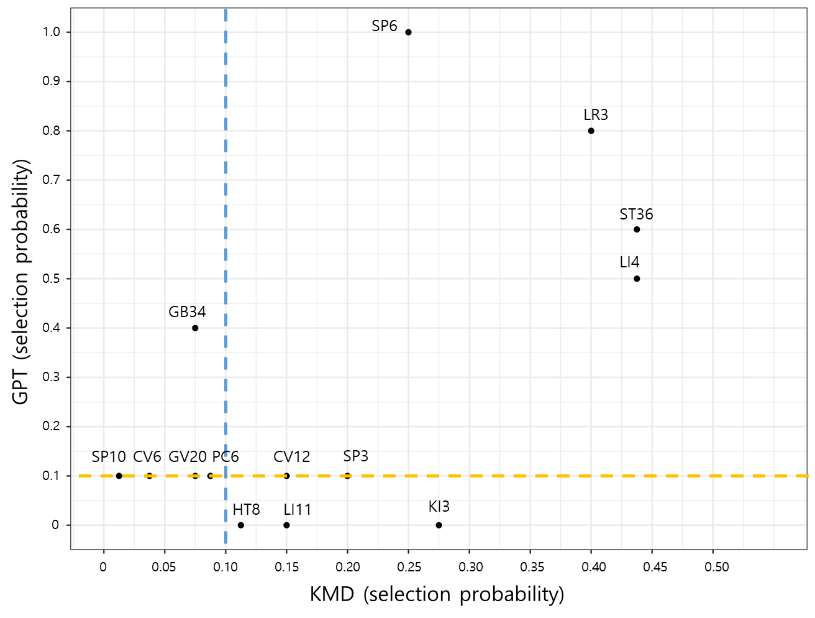


6. Case 06 (Chronic prostatitis)

| KMD  GPT | 0.10 | 0.20 | 0.30 | 0.40 | 0.50 |
| --- | --- | --- | --- | --- | --- |
| 0.10 | 0.29 | 0.29 | 0.24 | 0.24 | 0.24 |
| 0.15 | 0.13 | 0.13 | 0.13 | 0.13 | 0.13 |
| 0.20 | 0.33 | 0.33 | 0.33 | 0.33 | 0.33 |
| 0.25 | 0.00 | 0.00 | 0.00 | 0.00 | 0.00 |
| 0.30 | - | - | - | - | - |
| 0.35 | - | - | - | - | - |
| 0.40 | - | - | - | - | - |


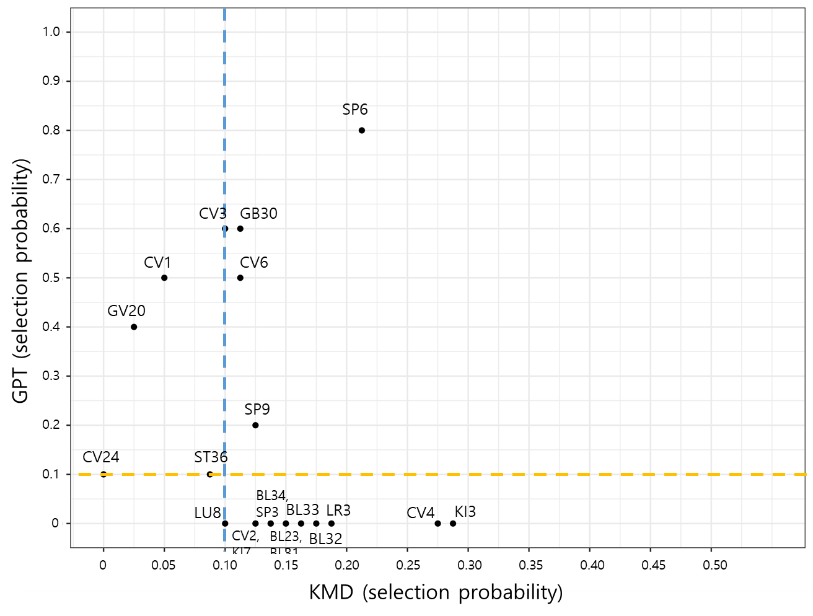


7. Case 07 (Panic disorder)

| KMD  GPT | 0.10 | 0.20 | 0.30 | 0.40 | 0.50 |
| --- | --- | --- | --- | --- | --- |
| 0.10 | 0.73 | 0.55 | 0.55 | 0.27 | 0.27 |
| 0.15 | 0.78 | 0.67 | 0.67 | 0.33 | 0.33 |
| 0.20 | 0.83 | 0.67 | 0.67 | 0.33 | 0.33 |
| 0.25 | 0.83 | 0.67 | 0.67 | 0.33 | 0.33 |
| 0.30 | 0.67 | 0.33 | 0.33 | 0.33 | 0.33 |
| 0.35 | 0.50 | 0.00 | 0.00 | 0.00 | 0.00 |
| 0.40 | 0.50 | 0.00 | 0.00 | 0.00 | 0.00 |


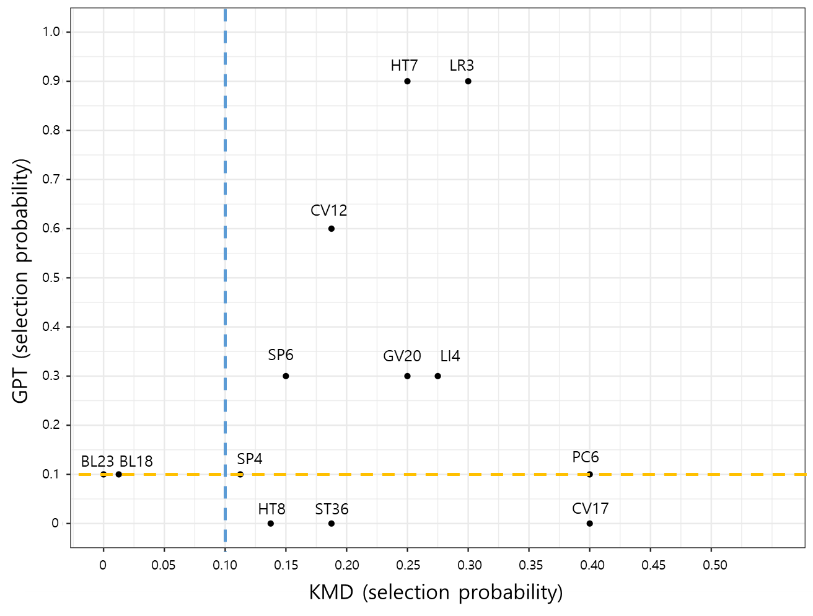


8. Case 08 (Intervertebral disc disorders)

| KMD  GPT | 0.10 | 0.20 | 0.30 | 0.40 | 0.50 |
| --- | --- | --- | --- | --- | --- |
| 0.10 | 0.27 | 0.27 | 0.27 | 0.18 | 0.18 |
| 0.15 | 0.38 | 0.38 | 0.38 | 0.25 | 0.25 |
| 0.20 | 0.60 | 0.60 | 0.60 | 0.40 | 0.40 |
| 0.25 | 0.75 | 0.75 | 0.75 | 0.50 | 0.50 |
| 0.30 | 0.50 | 0.50 | 0.50 | 0.50 | 0.50 |
| 0.35 | 0.00 | 0.00 | 0.00 | 0.00 | 0.00 |
| 0.40 | - | - | - | - | - |


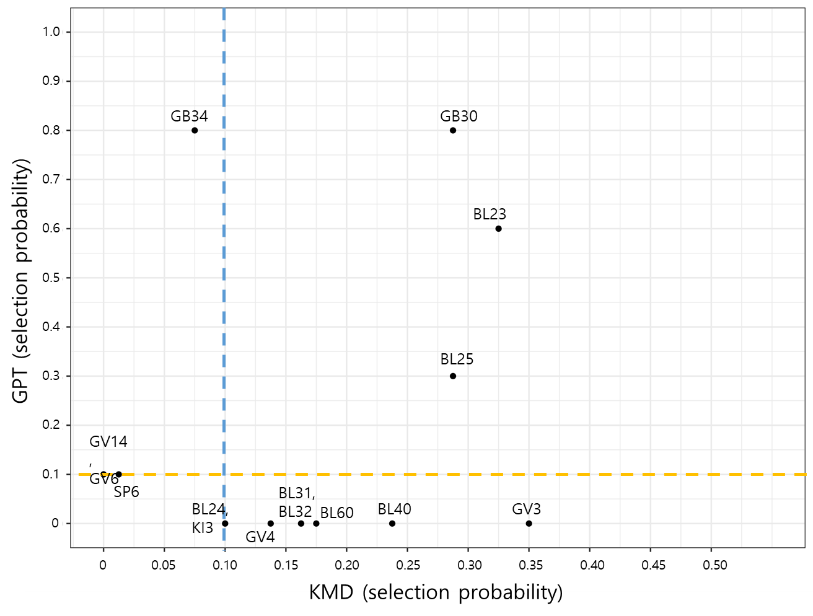


9. Case 09 (Fibromyalgia)

| KMD  GPT | 0.10 | 0.20 | 0.30 | 0.40 | 0.50 |
| --- | --- | --- | --- | --- | --- |
| 0.10 | 0.47 | 0.33 | 0.20 | 0.20 | 0.13 |
| 0.15 | 0.57 | 0.43 | 0.43 | 0.43 | 0.29 |
| 0.20 | 0.75 | 0.75 | 0.75 | 0.75 | 0.50 |
| 0.25 | 1.00 | 1.00 | 1.00 | 1.00 | 0.67 |
| 0.30 | - | - | - | - | - |
| 0.35 | - | - | - | - | - |
| 0.40 | - | - | - | - | - |


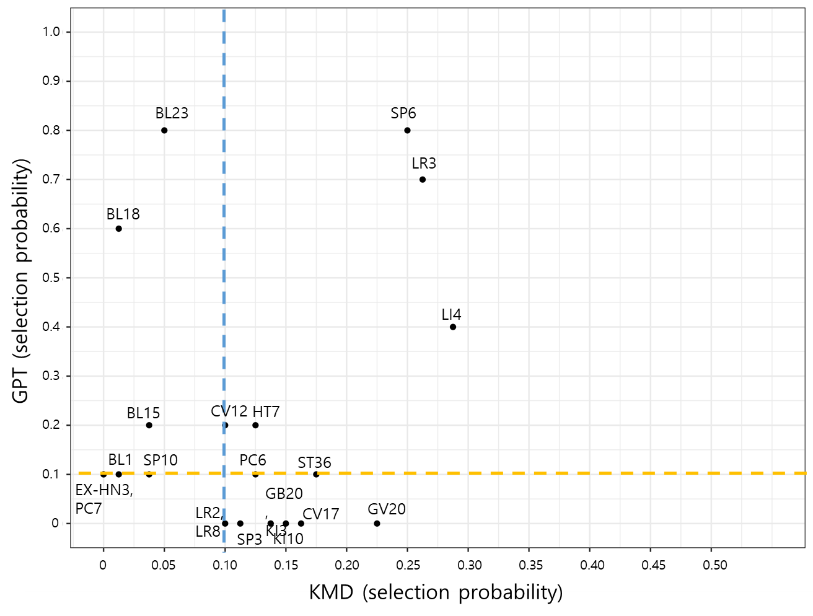


10. Case 10 (Puerperal disorder)

| KMD  GPT | 0.10 | 0.20 | 0.30 | 0.40 | 0.50 |
| --- | --- | --- | --- | --- | --- |
| 0.10 | 0.31 | 0.23 | 0.23 | 0.23 | 0.15 |
| 0.15 | 0.57 | 0.43 | 0.43 | 0.43 | 0.29 |
| 0.20 | 0.75 | 0.50 | 0.50 | 0.50 | 0.50 |
| 0.25 | 0.75 | 0.50 | 0.50 | 0.50 | 0.50 |
| 0.30 | 0.67 | 0.33 | 0.33 | 0.33 | 0.33 |
| 0.35 | 0.67 | 0.33 | 0.33 | 0.33 | 0.33 |
| 0.40 | 0.00 | 0.00 | 0.00 | 0.00 | 0.00 |


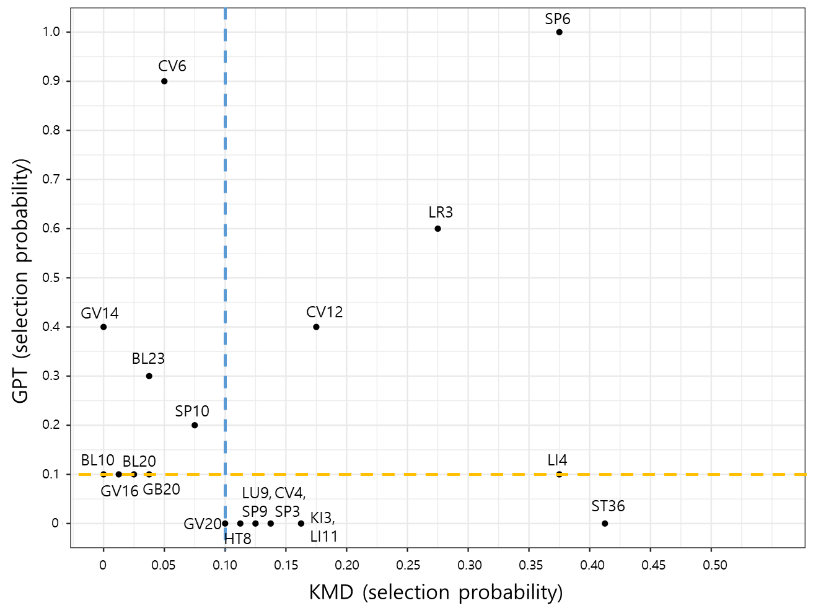

Supplement: Supplementary file 1 [file Supplementary_file_1.docx]
